# IL-21 is required for the maintenance and pathogenesis of murine Vγ4^+^ IL-17-producing γδT cells

**DOI:** 10.3389/fimmu.2023.1211620

**Published:** 2023-08-18

**Authors:** Junichi Ishikawa, Akira Suto, Kazuya Abe, Yuki Hayashi, Kensuke Suga, Shigeru Tanaka, Takahiro Kageyama, Arifumi Iwata, Kazumasa Suzuki, Kotaro Suzuki, Hiroshi Nakajima

**Affiliations:** ^1^ Department of Allergy and Clinical Immunology, Graduate School of Medicine, Chiba University, Chiba, Japan; ^2^ Institute for Advanced Academic Research, Chiba University, Chiba, Japan; ^3^ Chiba University Synergy Institute for Futuristic Mucosal Vaccine Research and Development (cSIMVa), Chiba, Japan

**Keywords:** IL-21, IL-17, γδT cells, Vγ4, experimental autoimmune encephalomyelitis

## Abstract

Murine IL-17-producing γδT (γδT17) cells are divided into two subsets: natural γδT17 (nγδT17) cells, whose development is restricted to the fetal thymus, and inducible γδT17 cells, which require antigen exposure for their IL-17 production and are presumed to develop from *Rorc* ^+^ *Il17a* ^-^ *CCR9* ^+^ immature γδT17 cells in the adult thymus and whose T cell receptor (TCR) is biased toward Vγ4. Although IL-23 is known to be involved in developing γδT17 cells, the roles of other cytokines, such as IL-21, which is involved in developing Th17 cells like IL-23, in the development, maintenance, and pathophysiology of γδT17 cells remain unknown. Here, we show that IL-21 is dispensable for the fetal thymic development of nγδT17 cells but is required for the peripheral maintenance of Vγ4^+^nγδT17 cells. Upon stimulation with γδTCR, IL-1 plus IL-21 induces the proliferation of Vγ4^+^nγδT17 cells via STAT3 as effectively as IL-1 plus IL-23. Using bone marrow chimeric mice, we demonstrated that immature γδT17 cells are produced *de novo* in the adult mice from donor adult bone marrow cells and that IL-21 is dispensable for their development. Instead, IL-21 is required to expand newly induced Vγ4^+^γδT17 cells in the periphery upon immunization. Finally, using adoptive transfer experiments of γδT17 cells, we found that IL-21 receptors on γδT17 cells are involved in maintaining Vγ4^+^γδT17 cells, subsequent infiltration of Th17 cells into the spinal cord, and exacerbation of experimental autoimmune encephalomyelitis. Collectively, IL-21 plays a vital role in the maintenance and pathogenesis of Vγ4^+^γδT17 cells.

## Introduction

γδT cells rapidly produce IL-17 and play crucial roles in infection and autoimmune diseases. Murine IL-17-producing γδT (γδT17) cells are divided into two subsets: natural γδT17 (nγδT17) cells and inducible γδT17 cells (1, 2). nγδT17 cells, whose TCR is biased toward Vγ4 and Vγ6 (Heilig and Tonegawa’s nomenclature) (3), can produce IL-17 without explicit induction of immune responses. Notably, their development is restricted to the embryonic thymus (4). In contrast to nγδT17 cells, inducible γδT17 cells are presumed to develop from the naïve compartment of γδT cells in adult lymph nodes (LNs) upon antigen stimulation and whose TCR is biased toward Vγ4 (5). Upon antigen stimulation, inducible γδT17 cells can acquire effector function without extensive clonal expansion and produce IL-17 within 60 hours. Recently, γδT cells that are positive for *Rorc* but negative for *Il17a* and *Il17f* were discovered in the immature CD24^+^ compartment of adult thymic γδT cells based on single-cell RNA sequence analysis. These immature γδT17 cells in the adult thymus are presumed to be a precursor of inducible γδT17 cells (6).

Upon antigen stimulation, γδT17 cells are also newly induced in LNs of Rag1-deficient (Rag1^-/-^) mice or TCRδ-deficient (TCRδ^-/-^) mice that are lethally irradiated and transplanted with wild-type bone marrow cells, even if mature CD90^+^ cells are depleted from these bone marrow cells (7–9). Given that these bone marrow chimeric (BMC) mice lack nγδT17 cells, these newly induced γδT17 cells in the BMC mice are called *de novo* γδT17 cells or bona fide γδT17 cells (7, 8). In this study, we call these γδT17 cells “newly induced” γδT17 cells.

Recent studies have shown that IL-23 is vital in expanding γδT17 cells in the periphery (7, 8). Nevertheless, because substantial numbers of γδT17 cells still exist in the LNs in mice lacking IL-23 receptors (8), cytokines other than IL-23 also seem involved in maintaining γδT17 cells. Since IL-23 uses STAT3 as a signaling molecule, other STAT3 users, such as IL-21, which induces Th17 cell differentiation (10), may be involved in maintaining γδT17 cells.

In this regard, previous studies have shown that γδT17 cells are decreased in the spleen and LNs of IL-21^-/-^ mice (10, 11). In contrast, Moser et al. have shown that γδT17 cells are increased in the lung and peritoneal cavity in IL-21 receptor-deficient (IL21R^-/-^) mice (12). Moreover, IL-21 has been shown to induce apoptosis of Vγ6^+^γδT17 cells (13). Since Vγ4^+^γδT17 cells are dominant in the spleen, whereas Vγ6^+^γδT17 cells are dominant in the lung and peritoneal cavity (14), IL-21 may be required for maintaining Vγ4^+^γδT17 cells but not Vγ6^+^γδT17 cells. However, the roles of IL-21 in the development, maintenance, and pathogenesis of Vγ4^+^γδT17 cells remain unknown.

We here investigated the roles of IL-21 in natural, immature, and newly induced Vγ4^+^γδT17 cells and found that IL-21 is not required for the development of Vγ4^+^nγδT17 cells in the fetal thymus and immature Vγ4^+^γδT17 cells in the adult thymus but is required for the maintenance of Vγ4^+^ γδT17 cells in the periphery. We also found that IL-21 signaling in γδT17 cells exacerbates experimental autoimmune encephalomyelitis (EAE).

## Methods

### Mice

C57BL/6 mice were purchased from CLEA Japan, Inc. (Tokyo, Japan). C57BL/6 Ly5.1 congenic mice were obtained from RIKEN BRC (Tsukuba, Japan). IL-21 receptor (IL-21R)-deficient (IL21R^-/-^) mice (15) were backcrossed over 8 generations onto C57BL/6 mice. TCRδ^-/-^ mice were obtained from the Jackson Laboratory (Bar Harbor, ME). All the mice were housed in microisolator cages under specific pathogen-free conditions. The Chiba University Animal Care and Use Committee approved animal procedures used in this study.

### Cell isolation and *in vitro* culture of γδT17 cells

RPMI 1640 medium containing 10% fetal calf serum, antibiotics, 1 x GlutaMAX (Gibco), 10 mM HEPES (Gibco), 1 mM sodium pyruvate (Gibco), 54 μM 2-mercaptoethanol, and 1 x non-essential amino acids (Gibco) was used for cell culture (complete γδT medium). For the purification of nγδT17 cells, B220^-^CD11b^-^TER119^-^CD4^-^CD8^-^ cells were purified from splenocytes and LN cells of naïve WT mice or IL21R^-/-^ mice using biotin-conjugated antibodies and MojoSort streptavidin nanobeads according to the manufacturer’s instructions (BioLegend, San Diego, CA), and then CD27^-^TCRγ/δ^+^ cells, previously defined as γδT17 cells in the periphery (16), were sorted using an SH800 cell sorter (Sony, Tokyo, Japan). 4 x10^3^ cells were stimulated with plate-bound anti-TCRγ/δ antibody (1 μg/ml) in the presence of anti-IFN-γ antibody (10 μg/ml) and indicated cytokines (IL-1β: 5 ng/ml, IL-23: 5 ng/ml, IL-21: 20 ng/ml) in a 96 well round-bottom plate for three days. Cells were then washed and cultured in a new well without anti-TCRγ/δ antibody in the presence of indicated cytokines for another three days.

γδT17 cell expansion culture from whole splenocytes was performed as described elsewhere (17) with minor modifications. In brief, 2.5 x10^5^ cells from the spleen and LNs were stimulated with plate-bound anti-TCRγ/δ antibody (1 μg/ml) in the presence of IL-1β (5 ng/ml) and IL-23 (5 ng/ml) in 96 well flat-bottom plates for three days. Cells were washed and cultured in a new well without anti-TCRγ/δ antibody in the presence of IL-1β and IL-23 for another three days. After washing, cells were cultured in the presence of IL-7 (20 ng/ml) for another three days.

### Reagents

Antibodies used in this study are listed in **Supplementary Table 1**. Recombinant murine IL-1β and IL-23 were purchased from R&D Systems (Minneapolis, MN), and recombinant murine IL-7 and IL-21 were purchased from BioLegend. STAT3 inhibitor (S3I-201) was purchased from Selleck Chemicals (Houston, TX).

### Staining of Vγ6^+^γδT17 and Vγ4^+^γδT17 cells

Vγ6 staining was performed as described elsewhere (18) with minor modifications. In brief, before staining of cell surface markers, cells were stained with anti-TCRγ/δ antibody (clone: GL3) (BioLegend), followed by 17D1 hybridoma supernatants that recognize both Vγ5 and Vγ6 TCR. FITC-conjugated mouse anti-rat IgM monoclonal antibody (MRM-47, BioLegend) was used to capture cells that were positively stained with 17D1 hybridoma supernatants. Anti-Vγ1 and Vγ5 TCR staining and other cell surface staining were performed along with FITC-conjugated mouse anti-rat IgM antibody.

### Apoptosis assay

Apoptotic cells were stained with Annexin V according to the manufacturer’s instruction (BD bioscience).

### Intracellular staining and flow cytometry analysis

To detect intracellular cytokines, cells were stimulated with PMA and ionomycin for 4 hours in the presence of BD GolgiPlug (BD Biosciences, Franklin Lakes, NJ). Intracellular cytokine and transcription factor staining were performed using an eBioscience Foxp3 transcription factor fixation/permeabilization kit (Thermo Fisher, Waltham, MA) as described previously (19). Cells were analyzed by FACS Canto II, FACS LSR Fortessa (BD Bioscience), or Novocyte Penteon (Agilent Technologies, Santa Clara, CA). FACS profiles were analyzed using the FlowJo software ver. 10.8 (BD Biosciences).

### Bone marrow chimeric mice

Bone marrow cells were isolated from CD45.1^+^ WT mice or CD45.2^+^ IL21R^-/-^ mice, and then CD90^+^ cells were depleted to remove mature αβ and γδ T cells using a biotin-conjugated anti-CD90.2 antibody (30-H12, BioLegend) and Mojosort streptavidin nanobeads (BioLegend). CD90^-^ bone marrow cells (total 1x10^7^ cells) were injected intravenously to lethally irradiated (950 rad) TCRδ^-/-^ mice. For generating mixed BMC mice, a mixture of CD90^-^ bone marrow cells (total 1x10^7^ cells) of CD45.1^+^ WT mice and CD45.2^+^ IL21R^-/-^ mice at a 1:1 ratio was injected intravenously to lethally irradiated TCRδ^-/-^ mice.

### Immunization with CFA and Ptx and induction of EAE

Mice were injected subcutaneously with complete Freund’s adjuvant (CFA), which is an emulsion of PBS, incomplete Freund’s adjuvant (IFA) (Chondrex), and M. tuberculosis extract H37 Ra (Difco) (PBS 100 μl, IFA 100 μl, and M. tuberculosis extract 0.4 mg/mouse) into the tail base, and then intraperitoneally injected with pertussis toxin diluted in PBS (200 ng/mouse) 6 hours and 48 hours after CFA administration. For the induction of EAE, MOG 35-55 peptide (MEVGWYRSPFSRVVHLYRNGK) was emulsified together with CFA (100 μg/mouse). The induction of EAE and scoring are described elsewhere (19, 20).

### Statistics

Statistical analyses were performed using GraphPad Prism ver.9 (GraphPad Software), and the results are described in each Figure legend. Data are summarized as mean ± SEM.

## Results

### IL-21 is dispensable for Vγ4^+^nγδT17 cells in the fetal thymus but is required for maintaining Vγ4^+^nγδT17 cells in the periphery in adult mice

**Figure 1:**
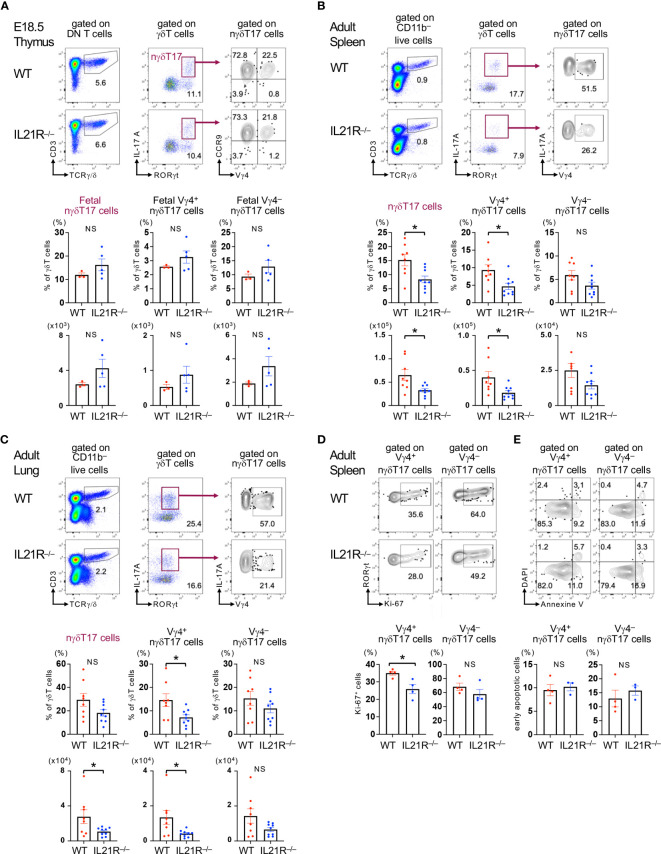
IL-21 is required for maintaining Vγ4^+^nγδT17 cells in adult mice. **(A)** Upper panels: Thymus of IL21R^-/-^ mice and littermate WT mice on embryonic day 18.5 was analyzed. Representative flow cytometric analyses of TCRγ/δ vs. CD3 on CD4^-^CD8^-^ (DN) T cells, RORγt vs. IL-17A on γδT cells, and CCR9 vs. Vγ4 on γδT17 (RORγt^+^IL-17^+^) cells are shown. Lower panels: The frequencies of RORγt^+^IL-17^+^CCR9^+^ fetal natural γδT17 (nγδT17) cells and Vγ4^+^ and Vγ4^-^ fetal nγδT17 cells among the total γδT cells and their absolute numbers are shown. n=3 for WT mice, n=5 for IL21R^-/-^ mice. **(B, C)** Upper panels: Spleen **(B)** and lungs **(C)** of naïve IL21R^-/-^ mice and littermate WT mice were analyzed at 8 weeks of age. Representative flow cytometric analyses of TCRγ/δ vs. CD3 on CD11b- live cells, RORγt vs. IL-17A on γδT cells, and Vγ4 vs. IL-17A on γδT17 cells are shown. Lower panels: The frequencies of RORγt^+^IL-17^+^nγδT17 cells, Vγ4^+^nγδT17 cells, and Vγ4^-^nγδT17 cells among the total γδT cells and their absolute numbers are shown. n=8-9, each. **(D)** Upper panels: Representative flow cytometric analyses of Ki-67 vs. RORγt on Vγ4^+^RORγt^+^ γδT cells and Vγ4^–^RORγt^+^ γδT cells in naïve WT and IL21R^-/-^ mice are shown. Lower panels: The frequencies of Ki-67^+^ cells among Vγ4^+^RORγt^+^γδT cells and Vγ4^–^RORγt^+^ γδT cells are shown. n = 4, each. **(E)** Upper panels: Representative flow cytometric analyses of Annexin V vs. DAPI on Vγ4^+^CD27^-^ γδT cells and Vγ4^-^CD27^-^ γδT cells in naïve WT and IL21R^-/-^ mice are shown. Lower panels: The frequencies of Annexin V^+^ DAPI^-^ early apoptotic cells among Vγ4^+^CD27^-^ γδT cells and Vγ4^-^CD27^-^ γδT cells are shown. n = 4 each NS, not significant, *p<0.05, unpaired t-test.

We first investigated the role of IL-21 in developing nγδT17 cells in the fetal thymus. IL-17A^+^RORγt^+^CCR9^+^γδT cells, which represent nγδT17 cells in the fetal thymus (6), and Vγ4^+^nγδT17 cells were comparable between IL21R^-/-^ mice and littermate WT mice (**Figure 1A**). As previously reported (10), nγδT17 cells were lower in the spleen in adult IL21R^-/-^ mice than in WT mice (**Figure 1B**). Notably, Vγ4^+^nγδT17 cells but not Vγ4^-^nγδT17 cells were significantly decreased in IL21R^-/-^ mice (**Figure 1B**). Inconsistent with a previous report showing increased lung γδT17 cells in IL21R^-/-^ mice (12), we found that the number of nγδT17 cells in the lung was decreased in IL21R^-/-^ mice as compared to WT mice (**Figure 1C**). Again, Vγ4^+^nγδT17 cells but not Vγ4^-^nγδT17 cells were decreased in the lungs of IL21R^-/-^ mice (**Figure 1C**). This discrepancy may be due to environmental factors because the lungs are rich in Vγ6^+^γδT17 cells, whose pool is affected by commensal bacteria and increases with age (14, 18). Meanwhile, the frequencies of Vγ4^+^nγδT17 cells and Vγ4^-^nγδT17 cells in the skin and large intestine were not significantly different between WT and IL21R^-/-^ mice (**Supplementary Figure 1**).

Given that IL-21 is constitutively produced by approximately 5% of splenic CD4^+^ T cells in naïve mice (21), we next analyzed proliferation and apoptosis markers of Vγ4^+^nγδT17 cells and Vγ4^-^nγδT17 cells in the spleen of naïve mice. The active proliferation marker Ki-67 positive cells were significantly decreased in Vγ4^+^nγδT17 cells but not in Vγ4^–^nγδT17 cells in naive IL21R^-/-^ mice compared with those in naïve WT mice (**Figure 1D**). Meanwhile, the proportion of annexin V^+^ DAPI^–^ apoptotic cells in each subset of IL21R^-/-^ mice did not differ from that of WT mice (**Figure 1E**). These findings suggest that IL-21 is dispensable for developing nγδT17 cells in the fetal thymus but is indispensable for the maintenance and/or proliferation of Vγ4^+^nγδT17 cells in the periphery, especially in the spleen and the lung.

### IL-21 together with IL-1 induces the proliferation of Vγ4^+^nγδT17 cells via STAT3 signaling

**Figure 2:**
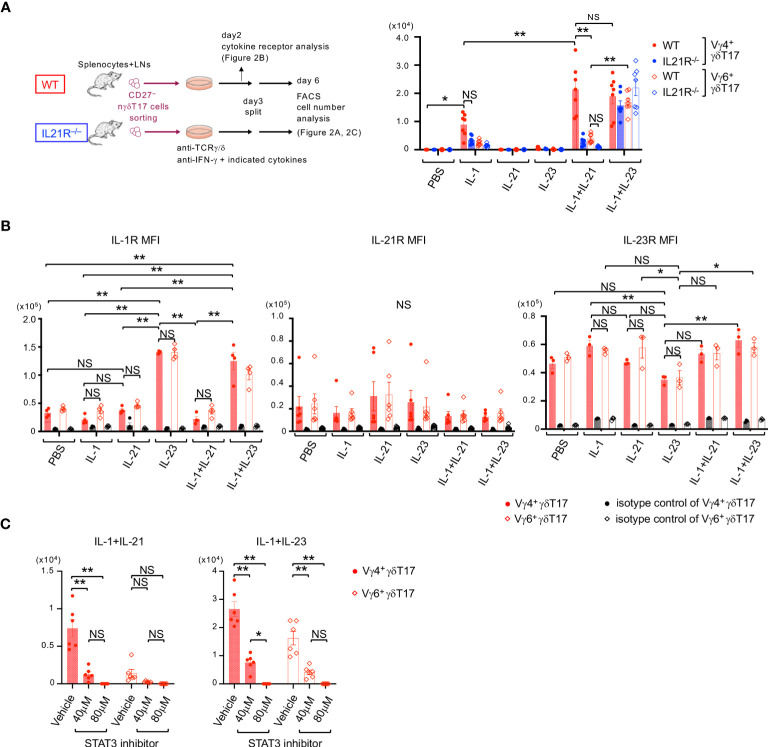
IL-21 together with IL-1 induces the proliferation of Vγ4^+^nγδT17 cells via STAT3. **(A)** Left panels: Schema of natural γδT17 cell culture. CD27^-^γδT cells were sorted from the spleen and LNs of naïve IL21R^-/-^ mice and WT mice, stimulated with anti-TCRγ/δ in the presence of anti-IFN-γ antibody and indicated cytokines, and subjected to flow cytometric analysis on day6. Right panels: The numbers of Vγ4^+^γδT17 cells and Vγ6^+^γδT17 cells after *in vitro* culture of natural γδT17 cells are shown. n=7, each. **p<0.01 by three-way ANOVA followed by Tukey’s multiple comparisons. NS: not significant. **(B)** CD27^-^γδT cells were sorted from the spleen and LNs of WT mice, stimulated with anti-TCRγ/δ in the presence of anti-IFN-γ antibody and indicated cytokines, and subjected to flow cytometric analysis on day 2. The mean fluorescence intensities (MFI) of IL-1R (left panels), IL-21R (middle panel), and IL-23R (right panel) on Vγ4^+^RORγt^+^γδT17 cells and Vγ6^+^RORγt^+^γδT17 cells are shown. n=4 for IL-1R, n=6 for IL21R, and n=3 for IL-23R. **p<0.01. Two-way ANOVA followed by Tukey’s multiple comparisons. **(C)** CD27^-^γδT cells were sorted from the spleen and LNs of WT mice, stimulated with anti-TCRγ/δ in the presence of anti-IFN-γ antibody and indicated cytokines, and either a STAT3 inhibitor (S3I-201, 40 or 80 μM) or vehicle. The numbers of Vγ4^+^γδT17 cells and Vγ6^+^RORγt^+^γδT17 cells after the culture are shown. n=6, each. **p<0.01. Two-way ANOVA followed by Tukey’s multiple comparisons. * p <0.05.

We next investigated the effect of IL-21 on the proliferation of nγδT17 cell subsets. For this purpose, CD27^-^ γδT cells, which represent γδT17 cells in the periphery (16), were purified from the spleen and lymph nodes of WT and IL21R^-/-^ mice (**Figure 2A**). At the start of the culture, the number of Vγ4^+^nγδT17 cells among CD27^-^ γδT cells was slightly decreased in IL21R^-/-^ mice (**Supplementary Figure 2**). Consistent with previous reports (7–9, 22), IL-1+IL-23 stimulation together with anti-TCRγ/δ induced approximately 10-fold proliferation of Vγ4^+^nγδT17 cells in WT mice and IL21R^-/-^ mice. Notably, IL-21 in combination with IL-1, but not IL-21 alone, induced the proliferation of Vγ4^+^nγδT17 cells as effectively as IL-1+IL-23 in WT mice but not in IL21R^-/-^ mice (**Figure 2A**). Importantly, Vγ6^+^nγδT17 cells, the major subset of Vγ4^–^γδT17 cells, did not significantly proliferate in response to IL-21+IL-1, but they responded well to IL-1+IL23 (**Figure 2A**), suggesting that the proliferative effect of IL-21+IL-1 is somewhat unique to Vγ4^+^nγδT17 cells.

We next examined cytokine receptor expression to determine how IL-21+IL-1 synergistically promotes Vγ4^+^γδT17 cell proliferation. Consistent with a previous report (22), IL-23 significantly upregulated the expression of IL-1R in both Vγ4^+^nγδT17 cells and Vγ6^+^nγδT17 cells (**Figure 2B**, left panel). These results may explain the synergistic effect of IL-1+IL23 on the proliferation of Vγ4^+^nγδT17 cells and Vγ6^+^nγδT17 cells. However, IL-21 did not significantly change the expression of IL-21R, IL-1R, and IL-23R in Vγ4^+^nγδT17 cells and Vγ6^+^nγδT17 cells (**Figure 2B**), suggesting that the alteration of cytokine receptor expression might not be the mechanism of different responses of Vγ4^+^nγδT17 cells and Vγ6^+^nγδT17 cells on IL-21+IL-1 stimulation. Moreover, the effect of IL-21+IL-1 on Vγ4^+^nγδT17 cell proliferation was not mediated through the production of IL-23, as the addition of an anti-IL-23 p19 neutralizing antibody did not have a significant effect (**Supplementary Figure 3**).

Given that IL-21 exerts its effect mainly through STAT3, PI3K, and MAPK pathway downstream of IL-21R (23) and that STAT3 is essential for IL-17 production from IL-23-stimulated Vγ4^+^ γδT17 cells (22), we next examined the role of STAT3 pathways. As shown in **Figure 2C**, a STAT3 inhibitor reduced the proliferation of IL-1+IL-23-stimulated Vγ4^+^nγδT17 cells and Vγ6^+^nγδT17 cells, as well as IL-21+IL-1-stimulated Vγ4^+^nγδT17 cells. Taken together, IL-21 induces the proliferation of IL-1-stimulated Vγ4^+^nγδT17 cells but not Vγ6^+^nγδT17 cells via the STAT3 pathway.

### IL-21 is dispensable for developing immature γδT17 cells in the adult thymus but is required for the expansion of Vγ4^+^γδT17 cells in the periphery upon antigen stimulation

**Figure 3:**
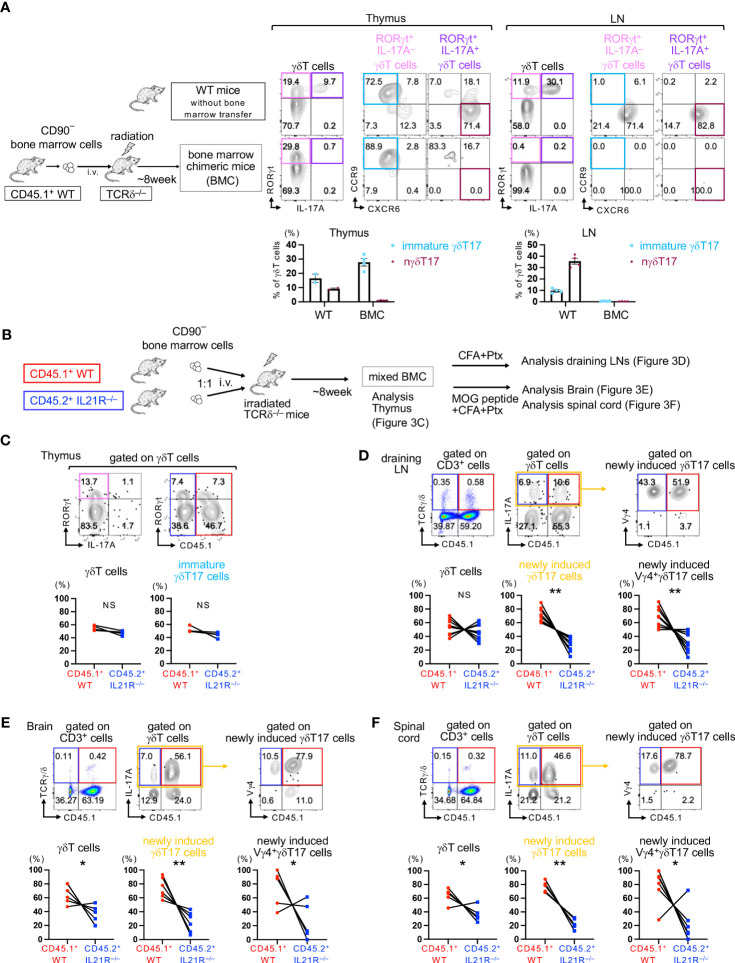
IL-21 is required for maintaining newly induced Vγ4^+^γδT17 cells. **(A)** Left panels: Schema of bone marrow chimeric (BMC) mice. CD90^–^ BM cells from CD45.1^+^ WT mice were intravenously injected into lethally irradiated TCRδ^-/-^ mice (BMC mice). Eight weeks after BM cell transfer, the thymus and LNs of BMC mice and untreated CD45.1^+^ WT mice (as a control) were analyzed. Upper panels: Representative flow cytometric analyses of IL-17A vs. RORγt gated on γδT cells and CXCR6 vs. CCR9 gated on RORγt^+^IL-17A^-^ γδT cells and RORγt^+^IL-17A^+^ γδT cells are shown. Lower panels: The frequencies of RORγt^+^IL-17A^-^CXCR6^-^CCR9^+^ immature γδT17 cells and RORγt^+^IL-17A^+^CXCR6^+^CCR9^-^ natural γδT17 cells among total γδT cells are shown. **(B)** Schema of mixed bone marrow chimeric (mBMC) mice. TCRδ^-/-^ mice were lethally irradiated (950 rad) and subsequently intravenously injected with a total of 1x10^7^ CD90^–^ BM cells from CD45.1^+^WT and CD45.2^+^IL21R^-/-^ mice at a 1:1 ratio. **(C)** Eight weeks after transfer, the thymus of steady-state mBMC mice was analyzed. Upper panels: Representative flow cytometric analyses of IL-17A vs. RORγt on γδT cells and CD45.1 vs. RORγt gated on γδT cells. IL-17A^-^RORγt^+^ immature γδT17 cells are surrounded by blue rectangles. Lower panels: The frequencies of CD45.1^+^WT and CD45.2^+^IL21R^-/-^ cells among total γδT cells and immature γδT17 cells. n=4, each. NS: not significant, one sample t-test compared the mean with a hypothetical value of 50. **(D)** mBMC mice were immunized with an emulsion of CFA followed by Ptx administration, and draining LNs were analyzed 14 days after immunization. Upper panels: Representative flow cytometric analyses of CD45.1 vs. TCRγ/δ on CD3^+^T cells, IL-17A vs. CD45.1 on γδT cells, and CD45.1 vs. Vγ4 gated on γδT17 cells. Lower panels: The frequencies of CD45.1^+^WT and CD45.2^+^IL21R^-/-^ cells among total γδT cells, γδT17 cells, and Vγ4^+^γδT17 cells. n=9, each. **p<0.01, one sample t-test compared the mean with a hypothetical value of 50. **(E, F)** mBMC mice were immunized with an emulsion of MOG peptide and CFA followed by Ptx administration, and the brain **(E)** and the spinal cord **(F)** were analyzed 21 days after immunization. FACS analyses were performed as shown in **(C)** n=6, *p<0.05, **p<0.01, one sample t-test compared the mean with a hypothetical value of 50. IL-17A^+^ newly induced γδT17 cells are surrounded by yellow rectangles **(D–F)**.

Next, we aimed to determine the role of IL-21 in the development of γδT17 cells that do not originate from the fetal thymus. Previous studies have shown that in lethally irradiated TCRδ^-/-^ mice or Rag1^-/-^ mice that received bone marrow (BM) cell transfer from WT mice, γδT17 cells originated from the fetal thymus are absent, but newly induced γδT17 cells can develop in the draining LNs (dLNs) following immunization (7–9). We employed these newly induced γδT17 cell systems to avoid the contamination of nγδT17 cells (**Figure 3A**).

First, we analyzed the development of γδT17 cells in the thymus of BMC mice before immunization. As expected, IL-17A^+^RORγt^+^CCR9^-^CXCR6^+^ nγδT17 cells were absent in the thymus of BMC mice but were present in the thymus of control naïve CD45.1 WT mice (**Figure 3A**). Importantly, IL-17A^-^RORγt^+^CCR9^+^CXCR6^-^ immature γδT17 cells could develop in the adult thymus even in TCRδ^-/-^ mice if these mice were received CD90^+^ mature T cells-depleted WT bone marrow cell transfer (**Figure 3A**). These results are consistent with previous reports showing that IL-17-producing γδT cells are absent in the thymus of BMC mice if IL-17 is used as a marker of γδT17 cells (7, 8). A recent study employing single-cell RNA sequencing has shown that immature γδT17 cells in the adult thymus are presumed to be a precursor of inducible γδT17 cells, which requires antigen stimulation to produce IL-17 (6). Therefore, both inducible γδT17 and newly induced γδT17 may originate from immature γδT17 cells, which can develop even in the adult thymus.

To determine the role of IL-21 in the development of immature γδT17 cells and newly induced γδT17 cells, we employed mixed BMC (mBMC) mice in which a mixture of CD45.1^+^WT and CD45.2^+^IL21R^-/-^ BM cells was injected intravenously into lethally irradiated adult TCRδ^-/-^ mice (**Figure 3B**). After the engraftment of donor cells, the frequencies of CD45.1^+^ T cells and CD45.2^+^ T cells in the spleen and lymph nodes were comparable (**Supplementary Figure 4**). The frequencies of γδT cells and IL-17A^-^RORγt^+^ immature γδT17 cells in the thymus were comparable between IL21R^-/-^ and WT cells, suggesting that the engraftment of γδT cells is equivalent and that IL-21 is indispensable for the development of immature γδT17 cells in the thymus (**Figure 3C**). However, upon stimulation with complete Freund’s adjuvant (CFA) and pertussis toxin (Ptx), the frequencies of newly induced γδT17 cells and Vγ4^+^ newly induced γδT17 cells in the dLNs were significantly lower in IL21R^-/-^ cells than in WT cells in mBMC mice (**Figure 3D**). Upon the induction of EAE, the frequencies of newly induced γδT17 cells and Vγ4^+^ newly induced γδT17 cells in the brain and spinal cord were significantly lower in IL21R^-/-^ cells in mBMC mice (**Figures 3E, F**). These findings indicate that IL-21 is dispensable for the development of immature γδT17 cells in the thymus but is required for the expansion of newly induced γδT17 cells and Vγ4^+^ newly induced γδT17 cells under inflammatory conditions.

### IL-21R on γδT17 cells is involved in exacerbating EAE

**Figure 4:**
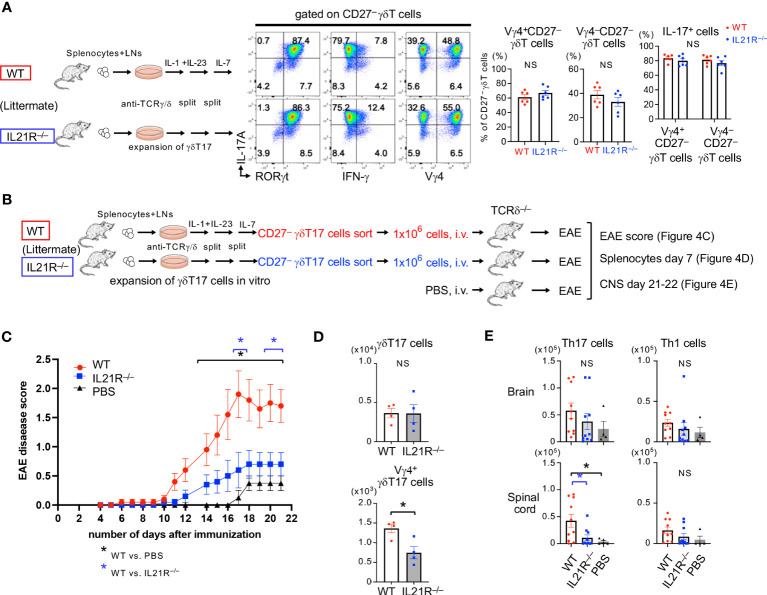
IL-21R on γδT17 cells is involved in exacerbating EAE. **(A)** Left panels: Splenocytes and LN cells from IL21R^-/-^ mice and littermate WT mice were cultured under γδT17 expansion conditions. Middle panels: Representative flow cytometric analyses of RORγt vs. IL-17A, IFN-γ vs. IL-17A, and Vγ4 vs. IL-17A on CD27^-^γδT17 cells are shown. Right panels: The frequencies of Vγ4^+^CD27^-^ γδT cells and Vγ4^-^CD27^-^ γδT cells among the CD27^-^ γδT cells and the frequencies of IL-17 producing cells among the Vγ4^+^CD27^-^ γδT cells and Vγ4^-^CD27^-^ γδT cells are shown. n = 6 each. NS: not significant, unpaired t-test. **(B)** Schema of experimental autoimmune encephalomyelitis (EAE) with adoptive transfer of γδT17 cells into TCRδ^-/-^ mice. Splenocytes and LN cells from IL21R^-/-^ mice and littermate WT mice were cultured under γδT17 cell expansion conditions for 9 days, as shown in **(A)**, and CD27^-^γδT17 cells were sorted by flow cytometry. TCRδ^-/-^ mice were intravenously injected with 1 x10^6^ WT γδT17 cells or IL21R^-/-^ γδT17 cells, or PBS (as a control) and immunized with an emulsion of MOG peptide and CFA followed by Ptx administration. **(C)** Mice were observed daily and scored for the clinical signs of EAE. n=10 for WT γδT17 cell-transferred mice and IL21R^-/-^ γδT17 cell-transferred mice, and n=4 for PBS-injected mice. *p<0.05, two-way ANOVA with repeated measures followed by Tukey’s multiple comparison test. *depicted in blue is WT γδT17 cell-transferred mice vs. IL21R^-/-^ γδT17 cell-transferred mice. *depicted in black is WT γδT17 cell-transferred mice vs. PBS-injected mice. **(D)** The frequencies of γδT17 cells and Vγ4^+^γδT17 cells in the spleen of IL21R^-/-^ γδT17 cell-transferred mice and WT γδT17 cell-transferred mice 7 days after immunization. n=4, each. *p<0.05, unpaired t-test. **(E)** The frequencies of Th17 cells and Th1 cells in the brain and spinal cord of IL21R^-/-^ γδT17 cell-transferred mice and WT γδT17 cell-transferred mice 21 days after immunization. n=10 for WT γδT17 cell-transferred mice and IL21R^-/-^ γδT17 cell-transferred mice, and n=4 for PBS-injected mice. *p<0.05, one-way ANOVA followed by Tukey’s multiple comparison test.

We finally examined the pathophysiological roles of IL-21R expressed on γδT17 cells in EAE. In this experiment, *in vitro*-expanded WT- or IL21R^-/-^CD27^-^γδT17 cells were prepared (**Figure 4A**) and intravenously injected into TCRδ^-/-^ mice, and EAE was induced in the recipient mice (**Figure 4B**). As shown in **Figure 4A**, after γδT17 cell expansion with IL-1+IL-23 and, subsequently, with IL-7 (17), most of the CD27^-^γδT17 cells were IL-17A^+^RORγt^+^ in WT and IL21R^-/-^ cells. The ratio of Vγ4^+^CD27^-^γδT17 cells to Vγ4^-^CD27^-^γδT17 cells and the capacity of IL-17 production in Vγ4^+^CD27^-^γδT17 cells and Vγ4^-^CD27^-^γδT17 cells were comparable between WT and IL21R^-/-^ cells (**Figure 4A**). After the adoptive transfer of γδT17 cells and the induction of EAE, IL21R^-/-^ γδT17 cell-transferred TCRδ^-/-^ mice showed lower EAE disease scores than WT γδT17 cell-transferred TCRδ^-/-^ mice (**Figure 4C**). TCRδ^-/-^ mice without γδT17 cell transfer (PBS) exhibited mild symptoms of EAE with a delayed onset, as previously reported (24).

Seven days after EAE induction, Vγ4^+^γδT17 cells but not total γδT17 cells in the spleen were significantly lower in IL21R^-/-^ γδT17 cell-transferred TCRδ^-/-^ mice than in WT γδT17 cell-transferred TCRδ^-/-^ mice (**Figure 4D**). Twenty-one days after EAE induction, although γδT17 cells were undetectable in the brain and spinal cord in all groups, Th17 cells in the spinal cord were significantly lower in IL21R^-/-^ γδT17 cell-transferred TCRδ^-/-^ mice than in WT γδT17 cell-transferred TCRδ^-/-^ mice (**Figure 4E**), consistent with a previous study showing that γδT17 cells amplify Th17 cell responses (25). These findings suggest that IL-21R on γδT17 cells maintains Vγ4^+^γδT17 cells, amplifies Th17 responses, and exacerbates EAE.

## Discussion

This study demonstrates the necessity of IL-21 in maintaining peripheral Vγ4^+^γδT17 cells. We show that IL-21 is dispensable for developing Vγ4^+^nγδT17 cells in the fetal thymus but is required for maintaining Vγ4^+^nγδT17 cells in the periphery in adult mice (**Figures 1**, **3**). We also show that IL-1+IL-21 induces the proliferation of anti-TCRγ/δ-stimulated Vγ4^+^γδT17 cells at a comparable level to IL-1+IL-23 (**Figure 2**). Through the adoptive transfer experiments of γδT17 cells, we show that IL-21 receptors expressed on γδT17 cells play a crucial role in the expansion of Vγ4^+^γδT17 cells, leading to the infiltration of Th17 cells into the central nervous system and the exacerbation of EAE (**Figure 4**). Thus, IL-21 has significant roles in maintaining Vγ4^+^γδT17 cells.

We demonstrated that the development of nγδT17 cells in the fetal thymus is unaffected in mice lacking IL-21R (**Figure 1A**). Because the production of IL-21 in the fetal thymus has not yet been reported, it is possible that nγδT17 cells in the fetal thymus are not exposed to IL-21. Accordingly, IL-21 could not play a role in developing nγδT17 cells in the fetal thymus.

By contrast, IL-21 is required to maintain Vγ4^+^nγδT17 cells in the periphery in adult mice (**Figures 1B, C**). IL-21 has been shown to be produced by thymic CD4^+^ T cells soon after birth, and these naturally occurring IL-21-producing CD4^+^ T cells are maintained by microbiota in the periphery (21) and seem to maintain the peripheral pool of Vγ4^+^nγδT17 cells in adult mice. Because Vγ4^+^γδT17 cells are resident in the subcapsular sinus (SCS) of lymph nodes and patrol around the CD169^+^ SCS macrophages and parenchyma (26, 27), they may be exposed to IL-21 in the T cell zone and stimulated by pathogen-derived molecules in the lymph flow and IL-1β produced by SCS macrophages in the LNs (28). Regarding IL-21-producing cells after immunization in the γδT17 cell-transfer experiment, the reduction of Vγ4^+^γδT17 cells in IL21R^-/-^ cells occurred on day 7 of immunization, prior to Th17 cell infiltration into the CNS, suggesting that Th17 cells may not be a source of IL-21 for maintaining Vγ4^+^γδT17 cells (**Figure 4**). Since we have previously shown that IL-21 is rapidly produced by naïve CD4^+^ T cells upon stimulation with anti-CD3ε and IL-6 (29), IL-21-producing cells that induce the proliferation of Vγ4^+^γδT17 cells may be TCR-and IL-6-stimulated CD4^+^ T cells or naturally occurring IL-21-producing CD4^+^ T cells (21).

IL-1 and IL-23 coordinately induce *in vitro* expansion of nγδT17 cells (16, 25, 30). IL-23 induces the expression of IL-1R, and IL-23, combined with IL-1β, induces the production of IL-17 synergistically (30). In this study, we found that IL-1+IL-21 induces the proliferation of anti-TCRγ/δ-stimulated Vγ4^+^nγδT17 cells as effectively as IL-1+IL-23 (**Figure 2A**). In contrast to IL-23, IL-21 did not increase the expression of IL-1R, and IL-1 did not affect the expression of IL-21R (**Figure 2B**). Thus, IL-1+IL-21 may induce the proliferation of Vγ4^+^nγδT17 cells through distinct mechanisms from IL-1+IL-23.

Since it has been demonstrated that IL-1 inhibits STAT3’s chromatin accessibility in a chondrosarcoma cell line (31), IL-21 may reverse this inhibition and induce the proliferation of IL-1-stimulated Vγ4^+^nγδT17 cells but not Vγ6^+^nγδT17 cells. In this regard, we found that STAT3 was essential for the proliferation of Vγ4^+^nγδT17 cells in either stimulation of IL-1+IL-21 or IL-1+IL-23 (**Figure 2C**). In our preliminary experiments, however, retrovirus-mediated forced expression of constitutively active STAT3 did not increase Ki-67 expression in Vγ4^+^nγδT17 cells under the stimulation with anti-TCRγ/δ + IL-1 (data not shown), suggesting that activation of STAT3 is necessary but is insufficient for the proliferation of Vγ4^+^nγδT17 cells. Additional research is required to identify the molecules that induce Vγ4^+^nγδT17 cell proliferation together with STAT3.

Regarding the role of IL-21 in another subset of nγδT17 cells, it has been shown that IL-21 induces apoptosis in Vγ6^+^nγδT17 cells (13). On the other hand, we found that Ki-67-positive proliferating cells were significantly decreased in Vγ4^+^nγδT17 cells but not in Vγ4^–^nγδT17 cells in IL21R^-/-^ mice compared with those in naïve WT mice (**Figure 1D**), whereas the proportion of apoptotic cells in each subset of IL21R^-/-^ mice did not differ from that of WT mice (**Figure 1E**). Consistently, IL-1+IL-21 did not induce the proliferation of anti-TCRγ/δ-stimulated Vγ4^-^nγδT17 cells, most of which are Vγ6^+^nγδT17 cells (**Figure 2A**). Thus, Vγ4^+^nγδT17 cells and Vγ6^+^nγδT17 cells respond differently to IL-21, mainly in proliferative responses. The mechanisms underlying the different responsiveness between Vγ4^+^nγδT17 cells and Vγ6^+^nγδT17 cells and the significance of this difference *in vivo* remain completely unknown, and this point also requires further investigation.

While nγδT17 cells arise during prenatal thymic development (4), inducible γδT17 cells are believed to develop from a naïve compartment of γδT cells in the lymph node in adult mice upon inflammation (2, 5). Indeed, newly induced Vγ4^+^γδT17 cells (called *de novo* or bona fide γδT17) develop in the draining LNs upon immunization in lethally irradiated adult TCRδ^-/-^ or Rag1^-/-^ mice whose hematopoietic environment is reconstituted with adult bone marrow cells even if CD90^+^ mature populations are depleted (7–9). The precursors of these newly induced Vγ4^+^γδT17 cells have been shown to be IL-23R^-^CD122^-^ *Rorc* ^+^ Vγ4^+^γδT17 cells, which emerged in the periphery in BMC mice (7), but the origin of these cells has not been identified. In this regard, a recent study using single-cell RNA sequence analysis has demonstrated that *Ccr9* ^+^ *Cxcr6* ^–^ *Rorc* ^+^ *Sox13* ^+^ *Maf* ^+^ *Il17a* ^–^ *Il17f* ^–^ immature γδT17 cells found in the adult thymus presumed to be the precursor of newly induced γδT17 cells (6). Consistent with previous reports, we found that RORγt^+^CXCR6^+^IL-17^+^ mature γδT17 cells were absent, but RORγt^+^CCR9^+^IL-17^–^ immature γδT17 cells developed in the thymus of BMC mice (**Figure 3A**). Therefore, BM cells could differentiate into immature γδT17 cells in adult mice, but the maturation is arrested at the RORγt^+^ IL-17^–^ immature state. It is plausible that these immature γδT17 cells migrate to lymph nodes and mature into newly induced γδT17 upon immunization. By breeding photoconvertible protein transgenic mice with Indu-Rag1 mice (4), in which the Rag1 gene can be turned on in adult mice by tamoxifen, it will be possible to find out if immature γδT17 cells made in the adult thymus can move to inflamed lymph nodes and turn into newly induced γδT17 cells.

IL21R deficiency also reduced the infiltration of newly induced Vγ4^+^γδT17 cells into the brain and the spinal cord (**Figures 3E, F**). Since IL-21 is involved in the upregulation of CX3CR1 expression on Vγ4^+^γδT17 cells in the draining LN of CFA-immunized mice (32) and CX3CR1-expressing cells accumulate in the inflammatory brain lesions of EAE (33), IL-21 may also play a role in the recruitment of Vγ4^+^γδT17 cells into the brain and the spinal cord via CX3CR1 induction. Consistently, we found that IL-21R deficiency in γδT17 cells ameliorated the severity of EAE. Since the deficiency of γδT cells reduces the severity of EAE and γδT17 cells increase susceptibility to EAE by amplifying Th17 cell responses (24, 25), it is plausible that IL-21R deficiency in γδT17 cells reduced the severity of EAE via the reduction of Th17 cells.

In conclusion, IL-21 plays a vital role in the maintenance and pathogenesis of Vγ4^+^γδT17 cells. Our results add new insight into the mechanisms of IL-21-mediated pathogenesis of autoimmune diseases and the development and maintenance of Vγ4^+^γδT17 cells.

## Author contributions

All authors were involved in drafting the article and approved the final version to be published. AS has full access to all data in the study and takes responsibility. Study conception and design: JI, AS, and HN. Performed research: JI, AS, KA, YH, KeS, and TK. Statistical analysis: JI, AS, ST, and AI. Manuscript preparation: JI, AS, ST, TK, AI, KaS, KoS, and HN.
