# Supplementary figures and images for: IL-21 is required for the maintenance and pathogenesis of murine Vγ4+ IL-17-producing γδT cells
Source: Front Immunol. 2023 Aug 18;14:1211620. doi: 10.3389/fimmu.2023.1211620 (PMC10473412; doi:10.3389/fimmu.2023.1211620)

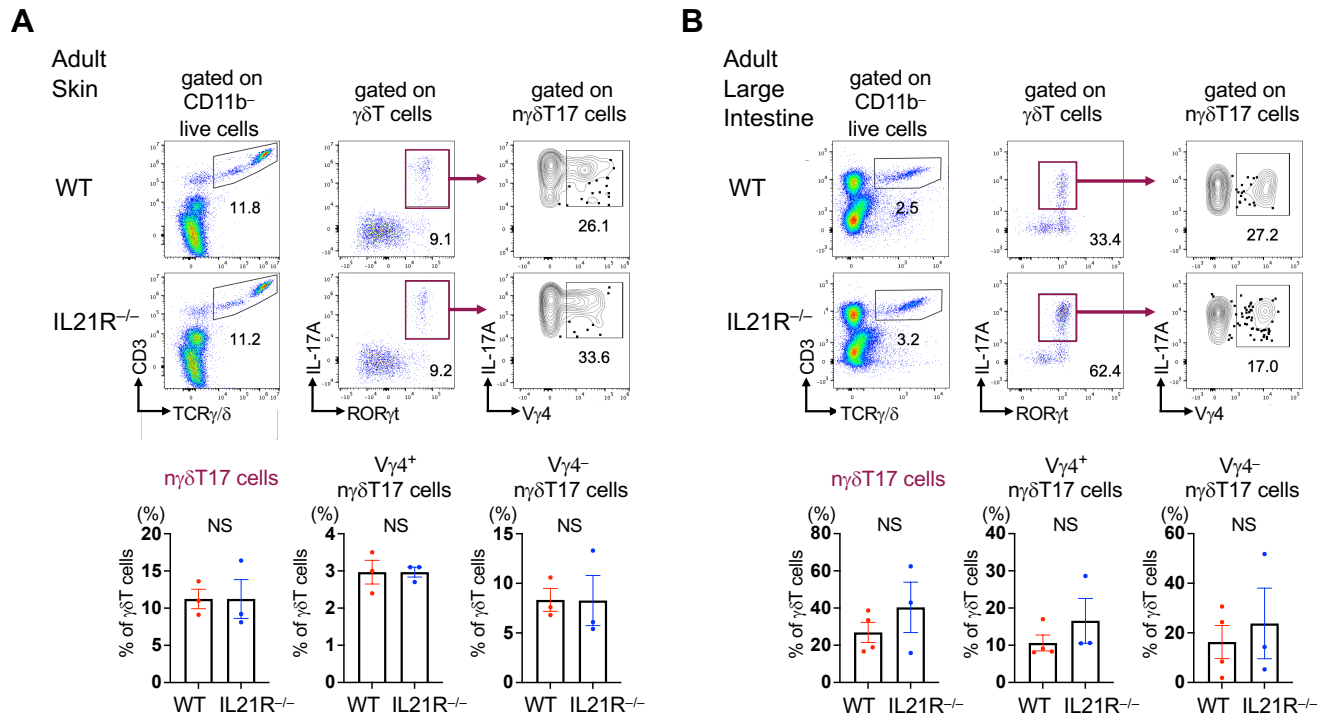

Supplementary Figure 2

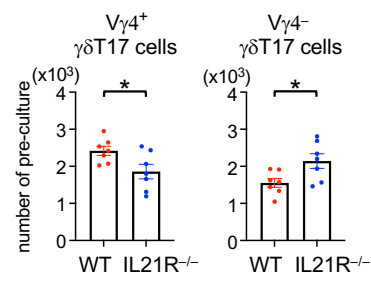

Supplementary Figure 3

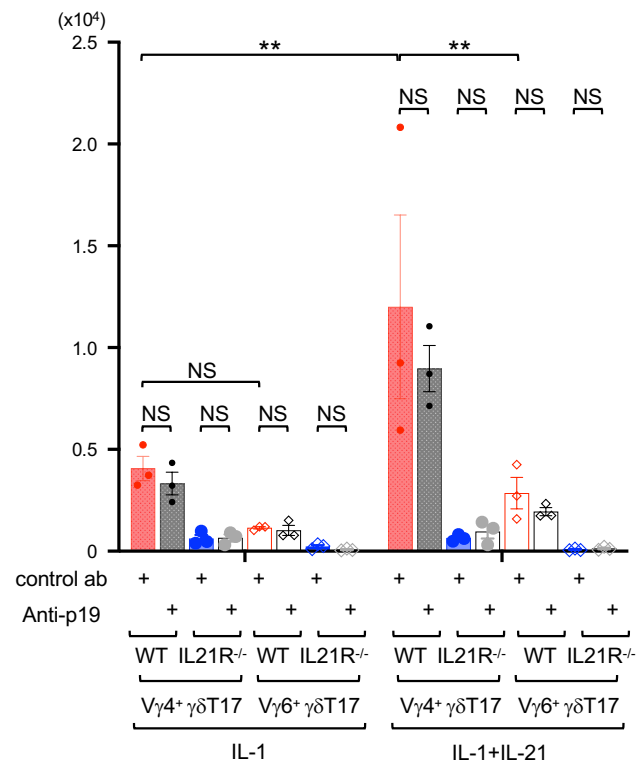

Supplementary Figure 4

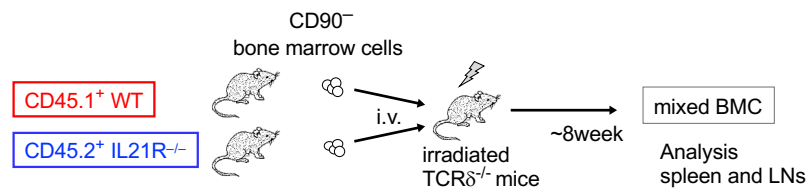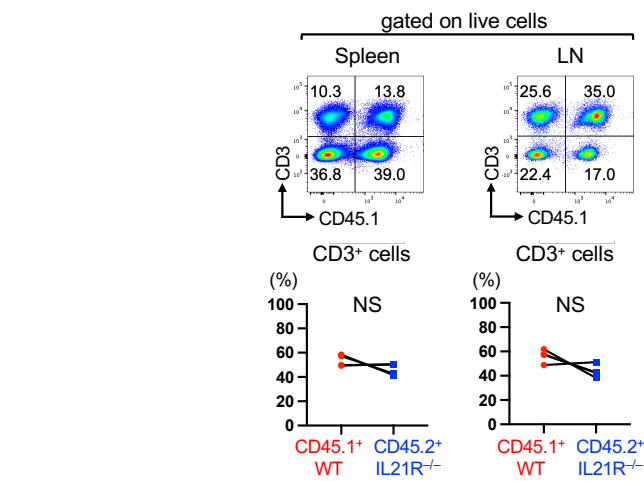

Supplement: Supplementary file 1 [file DataSheet_1.pdf]
