# Supplementary material for: IL-21 is required for the maintenance and pathogenesis of murine Vγ4+ IL-17-producing γδT cells
Source: Front Immunol. 2023 Aug 18;14:1211620. doi: 10.3389/fimmu.2023.1211620 (PMC10473412; doi:10.3389/fimmu.2023.1211620)
Supplement: Supplementary file 2 [file DataSheet_2.doc]

**Supplementary information**

**Supplementary Table 1. Antibodies and reagent used in this study**

| Antibodies | conjugate | Clone | Vendor |
| --- | --- | --- | --- |
| CD3 | AF700 | 17A2 | BioLegend |
| CD3ε | AF700 | 145-2C11 | BD Biosciences |
| CD4  CD8  CD11b  CD19  B220  TER119  CD90.2  CD27 | BV510, BV785, Biotin  BV711, PerCP, Biotin  APC ef780, PerCP, Biotin  PerCP  Biotin  Biotin  Biotin  BV421 | RM4-5  53-6.7  M1/70  1D3  RA3-6B2  TER-119  30-H12  LG.3A10 | BD Biosciences  BD Biosciences  Thermo Fisher  BD Biosciences  BioLegend  BioLegend  BioLegend  BioLegend |
| CD45.1 | BV605, BUV737 | A20 | BioLegend |
| TCRγ/δ  Vγ2* (Vγ4)  Vγ1.1* (Vγ1)  Vγ3* (Vγ5)  Ki-67  Annexin V | BV711, BV421, APC, none  PECy7, BUV615  APC  APC  BV510  PE | GL3  UC3-10A6  2.11  536  B56  N/A | BioLegend  BD Biosciences  BioLegend  BioLegend  BD Biosciences  BD Biosciences |
| CXCR6  CCR9 | FITC  PE, PerCP eF710 | SA051D1  CW1.2 | BioLegend  Thermo Fisher |
| IFN-γ  IL-17A | BV510, none  BV786, APC, PE | XMG1.2  TC11-18H10.1 | BioLegend  BD Biosciences |
| RORγt  IL-1R | AF647, BV421  PE | Q31-378  JAMA-147 | BD Biosciences  BioLegend |
| IL-21R  IL-23R  IL-23 (p19) | APC  PE  none | 4A9  12B2B64  MMp19B2 | BioLegend  BioLegend  BioLegend |

*TCR Vγ antibodies shown in this table use Garman’s nomenclature (1).

1. R. D. Garman, P. J. Doherty and D. H. Raulet: Diversity, rearrangement, and expression of murine T cell gamma genes*. Ce*ll, 45(5), 733-42 (1986) doi:10.1016/0092-8674(86)90787-7

**Supplementary Methods**

**Isolation of lymphocytes from back skin**

Back skin (4.5 cm^2^) was floated with the dermis side down and incubated in complete RPMI 1640 medium containing dispase II (5 mg/ml, Fujifilm Wako, Osaka, Japan) for 1 hour at 37 ℃. The skin was cut into small pieces and incubated in complete RPMI 1640 medium containing collagenase type 2 (1.6 mg/ml, Worthington, Lakewood, NJ) and hyaluronidase (1.2 mg/ml, Sigma-Aldrich, St Louis, MO) for 1 hour at 37℃. The skin tissues were dissociated using gentleMACS (Miltenyi Biotec, Auburn, CA) and separated into a single-cell suspension by passing through 100 μm, 50 μm, and 30 μm nylon cell strainers in order.

**Isolation of lamina propria lymphocytes from the large intestine**

Colonic tissues were treated with HBSS containing 1 mM dithiothreitol and 20 mM EDTA at 37℃ for 20 min to remove epithelial cells. The tissues were then minced and dissociated with Liberase TL (60 μg/ml) (Roche Diagnostics) and DNase I (0.1 mg/ml) (Wako Pure Chemical Industries) in RPMI 1640 medium (Sigma-Aldrich) at 37 ℃ for 30 min to obtain single-cell suspensions. After filtering, the single-cell suspensions were washed with 2% FCS in RPMI 1640 and subjected to purification by a debris removal solution (Miltenyi Biotec).

**Supplementary Figure legends**

**Supplementary Figure 1. nγδT17 cells in the skin and large intestine**

(**A, B**) Upper panels: Skin (**A**) and large intestine (**B**) of naïve IL21R^-/-^ mice and littermate WT mice were analyzed at 8 weeks of age. Representative flow cytometric analyses of TCRγ/δ vs. CD3 on CD11b^-^ live cells, RORγt vs. IL-17A on γδT cells, and Vγ4 vs. IL-17A on γδT17 cells are shown. Lower panels: The frequencies of RORγt^+^IL-17^+^nγδT17 cells, Vγ4^+^nγδT17 cells, and Vγ4^-^nγδT17 cells among the total γδT cells are shown. (**A**) n = 3 each. (**B**) n = 4 for WT mice and n = 3 for IL21R^-/-^ mice. NS: not significant, unpaired t-test.

**Supplementary Figure 2. Number of Vγ4^+^nγδT17 cells and Vγ4^-^nγδT17 cells at the start of culture**

Number of Vγ4^+^nγδT17 cells and Vγ4^-^nγδT17 cells in CD27^-^ nγδT17 cells from WT mice and IL21R^-/-^ mice at the start of culture in Figure 2A. n = 7. *p<0.05, unpaired t-test.

**Supplementary Figure 3.** **The effect of IL-21+IL-1 on Vγ4^+^nγδT17 cell proliferation is not mediated through the production of IL-23**

CD27^-^γδT cells sorted from naïve IL21R^-/-^ mice and WT mice were cultured under IL-1 or IL-21 + IL-1 conditions as described in the legend of Figure 2A in the presence of an anti-IL-23 (p19) neutralizing antibody or isotype-matched control antibody. The number of Vγ4^+^γδT17 cells and Vγ6^+^γδT17 cells after in vitro culture of natural γδT17 cells is shown. n=3, each. **p<0.01 by three-way ANOVA followed by Tukey’s multiple comparisons. NS: not significant.

**Supplementary Figure 4. The frequencies of CD45.1^+^ T cells and CD45.2^+^ T cells in the spleen and lymph nodes of mBMC mice**

Upper panels: Representative flow cytometric analyses of CD45.1 vs. CD3 gated on live cells in the spleen and LNs. Lower panels: The frequencies of CD45.1^+^WT and CD45.2^+^IL21R^-/-^ cells among total T cells in the spleen and LNs. n=4, each. NS: not significant, one sample t-test compared the mean with a hypothetical value of 50.
